# Perceived Parental Involvement Decreases the Risk of Adolescent Depression

**DOI:** 10.31083/AP46110

**Published:** 2025-08-28

**Authors:** Xuerong Liu, Wei Li, Jingyu Lei, Xiaodi Han, Qiongzhi Zhang, Qianyu Zhang, Jie Gong, Jingxuan Zhang, Zhiyi Chen, Zhengzhi Feng

**Affiliations:** ^1^Experimental Research Center of Medical and Psychological Science, School of Psychology, Third Military Medical University, 400038 Chongqing, China; ^2^Psychology Department, Nanchong Psychosomatic Hospital, 637000 Nanchong, Sichuan, China

**Keywords:** perceived parental involvement, adolescent, depression, linear mixed-effect models (LMM), latent profile analysis (LPA)

## Abstract

**Objective:**

To tailor culturally sensitive interventional strategies for safeguarding adolescents’ mental health, this study investigated the role of perceived parental involvement in predicting depressive symptoms among Chinese adolescents, considering family socioeconomic status (SES).

**Methods:**

A cluster convenience sampling method recruited 21,818 participants from 48 middle schools across 29 provinces in China. The perceived parental involvement (PPI) Scale and the Chinese version of the center for epidemiologic studies depression scale (CES-D) assessed parental involvement and depressive symptoms, respectively. Data analysis employed linear mixed-effect models (LMM) and latent profile analysis (LPA).

**Results:**

The results indicated that 35.26% of adolescents exhibited subclinical depressive symptoms. LMM analysis revealed that higher perceived parental involvement scores, particularly emotional involvement, significantly predicted lower CES-D scores (*β* = –0.45, *p* < 0.001). LPA identified three distinct family factors profiles, with the “High SES-High PPI” group showing the lowest depression scores.

**Conclusion:**

The findings underscore the protective benefits of perceived parental involvement, especially emotional support, in mitigating depressive symptoms among adolescents. Specifically, adolescents from families with both high SES and high parental involvement exhibited the lowest levels of depressive symptoms, suggesting that interventions should focus on enhancing emotional support and addressing socioeconomic disparities to effectively reduce adolescent depression.

## Main Points

1. Protective Role of Parental Involvement: The study revealed that perceived parental involvement, particularly in the emotional domain, significantly reduces the risk of depression in Chinese adolescents, with a strong negative predictive effect on depressive symptoms.

2. Broad Reach and Impact: Conducted across 29 provinces with over 21,000 participants, the study provides a comprehensive view of how family factors influence adolescent mental health, emphasizing the importance of considering both parental involvement and socioeconomic status in depression prevention strategies.

3. Distinct Family Profiles: Through latent profile analysis, the study identified three distinct family profiles that significantly predict adolescent depression levels, with the “High SES-High PPI” group showing the lowest depression scores.

4. Implications for Mental Health Interventions: The findings stress the importance of developing interventions focused on strengthening emotional bonds within families to mitigate depressive symptoms among adolescents, especially in the context of varying socioeconomic backgrounds.

## 1. Introduction

Adolescent depression is a significant global public health issue, with approximately 34% of adolescents worldwide showing symptoms [1]. The severity and prevalence of this condition cannot be ignored. It is evident that disentangle which factors contribute to adolescent depression is crucial for informing effective early interventions and public policymaking. To do so, the Attachment Theory provides a compelling conceptual framework for this study, where it underlined that secure and supportive attachment relationships, particularly with primary caregivers (e.g., parents), are leading components for developing emotional resilience and psychological well-being to decrease risks of mental health problems [2, 3]. Recently, with the rapid development of the socio-economic landscape and acceleration of the pace of life, in China, adolescents are increasingly facing academic pressure [4], family expectations [5], and challenges in social adaptation [6, 7]. These factors collectively contribute to the rising incidence of depressive symptoms among adolescents. The prevalence of adolescent depression is particularly concerning, provoking widespread discussion across various Chinese social sectors [8, 9]. Research indicates that adolescent depression not only affects their current quality of learning and life but may also have long-term negative impacts on their adult mental health [10]. Consequently, identifying and understanding the key factors influencing adolescent depression is of paramount importance.

Family environment is a critical factor in adolescent mental health as it provides essential emotional support and value orientation [11]. Parental involvement, encompassing material support, emotional care and spiritual guidance, plays a significant role in adolescent psychological development [12]. According to this domain-specific theoretical model, parental involvement—in its various forms—fosters a secure base for adolescents to explore their world, encouraging emotional stability and reducing vulnerability to depression [2]. Research shows that active parental involvement can alleviate depressive symptoms and promote mental health in adolescents [13]. For example, parental support and encouragement in academics can enhance adolescent self-confidence and sense of achievement, while emotional understanding and communication can help reduce feelings of loneliness and anxiety.

However, parental involvement is not a monolithic construct; it encompasses involvement in multiple dimensions, including emotional, social, life, and academic dimensions. Each dimension may exert distinct influences on adolescent depression. For example, emotional involvement, characterized by warmth, empathy and open communication, may directly alleviate depressive symptoms by fostering a sense of security and belonging [14, 15]. Similarly, academic involvement, such as helping with homework or monitoring academic progress, might reduce stress related to school performance but may also exacerbate depressive symptoms if perceived as overcontrolling or excessively demanding [16]. Further, social involvement, such as facilitating peer interactions or extracurricular activities, may enhance adolescent social adaptation, while life involvement, including guidance on daily routines, may promote stability and reduce stress [17, 18]. Such nuanced effects highlight the need to differentiate between types of parental involvement when examining their impact on adolescent mental health.

The characteristics and influencing factors of parental involvement among Chinese adolescents have not been thoroughly studied. The family structure and cultural background in China differ significantly from those in Western countries, which may lead to variations in the methods and effects of parental involvement [19]. In China, traditional family values emphasize strict discipline and high expectations from parents towards their children, which may influence the nature and extent of parental involvement [20, 21]. With rapid socio-economic development, many families face the reality of dual-income households, limiting the time and energy parents can devote to their children, potentially affecting their level of involvement. In the Chinese sociocultural contexts, both sharply societal pressures to education and rapid socioeconomic changes created unique challenges for adolescents, inevitably increasing risks of encountering mental health problems, particularly in depression.

While the connection between parental involvement and adolescent mental health is well-documented, much of the research has focused on Western contexts, overlooking cultural nuances that may affect these dynamics in China. Often, parental involvement is viewed as a single entity, without differentiating its various sub-dimensions and their specific impacts on adolescent mental health. This study hypothesizes that perceived parental involvement, along with its distinct dimensions, will differentially predict levels of adolescent depression.

Socioeconomic status (SES) is another critical factor influencing adolescent depression and family dynamics. SES is a multidimensional construct typically measured by parental education level, subjective economic status and family income (e.g., annual household income). In the Chinese context, SES manifests uniquely due to the nation’s rapid socio-economic transformation and deeply rooted cultural values. For example, families with higher SES often prioritize academic achievement and invest heavily in extracurricular activities and private tutoring, reflecting the cultural emphasis on education as a pathway to upward mobility [22]. Conversely, lower-SES families may focus more on providing emotional support and fostering resilience in their children, compensating for limited material resources [23]. These cultural characteristics suggest that the interaction between SES and parental involvement may vary significantly across different economic levels, influencing adolescent mental health outcomes in distinct ways.

Interaction effects between SES and parental involvement warrant further exploration. For instance, while high-SES families may leverage their financial and educational advantages to provide structured academic support, this could inadvertently increase pressure on adolescents, potentially exacerbating depressive symptoms if expectations are perceived as overwhelming [24]. Alternatively, in low-SES families, active emotional involvement and effective communication may serve as protective factors, mitigate the adverse effects of resource scarcity and reduce the risk of depression [25]. Understanding these nuanced dynamics is essential for developing culturally sensitive interventions tailored to families from diverse socioeconomic backgrounds.

Although existing research has highlighted the impact of SES on adolescent depression, the mechanisms through which parental involvement and family SES jointly influence adolescent depression remain unclear. Theoretically, active parental involvement may mitigate some of the negative effects associated with low SES. For instance, even in resource-limited settings, parents can provide adolescents with a sense of psychological security and belonging through emotional support and effective communication, thereby reducing the risk of depression [26]. Therefore, exploring the combined effects of parental involvement and family SES is crucial for understanding the complex etiology of adolescent depression.

Based on these issues, this study aims to explore how different dimensions of perceived parental involvement predict depression among Chinese adolescents. Additionally, the research will examine the interaction between family SES and parental involvement to identify distinct familial characteristics and their associations with depressive symptoms. Through this analysis, the study seeks to provide a more comprehensive perspective on the influence of familial factors on adolescent depression. Based on this well-established theoretical assumptions, given the potential protective role of parental support, investigating the specific nexus between parental involvements and depression can inform culturally tailored interventions to mitigate mental health risks.

## 2. Materials and Methods 

### 2.1 Participants 

**Fig. 1.**
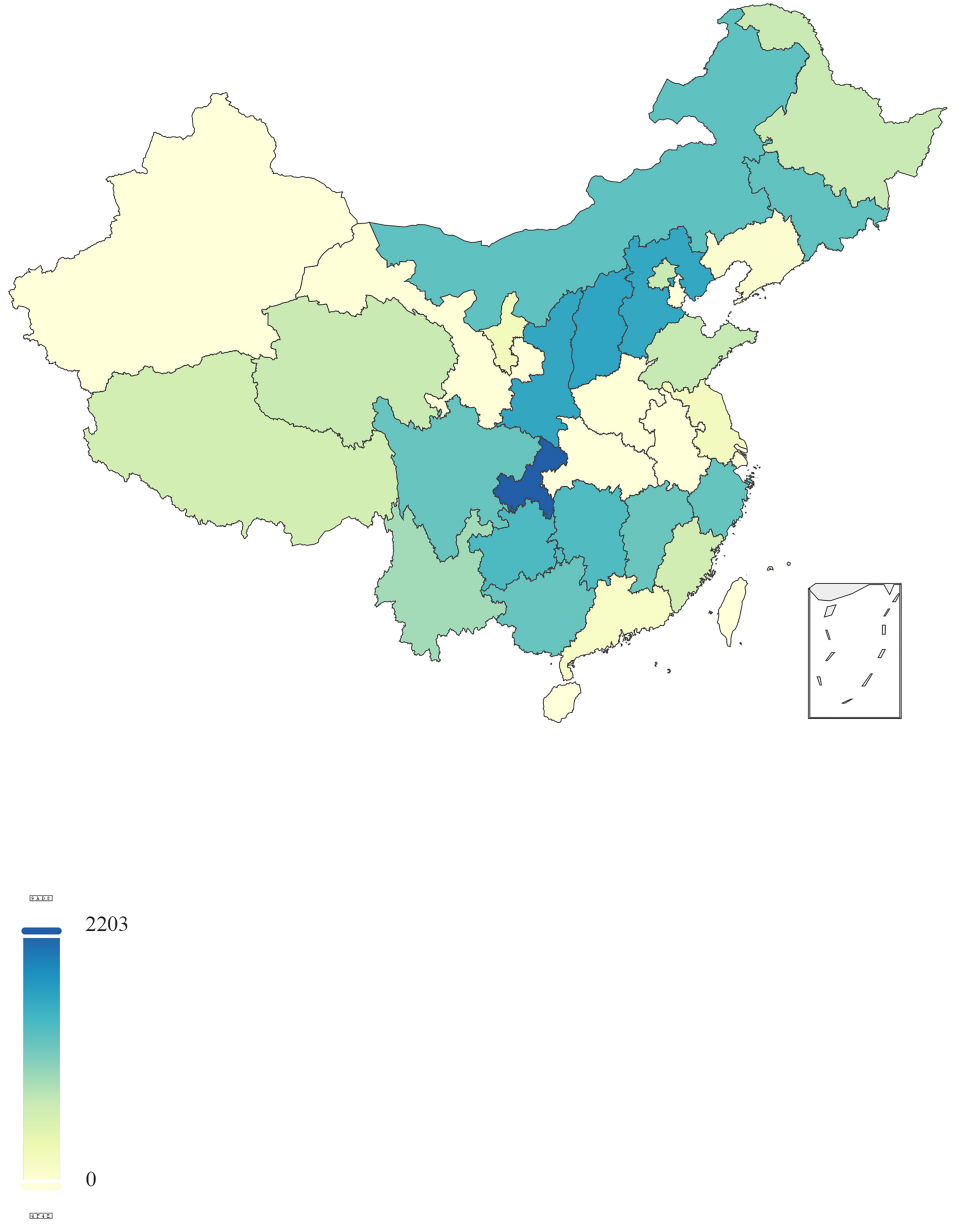
**Geographical distribution of sampled middle schools across China**.

This large-scale, nationwide study employed a cluster convenience sampling method, encompassing a diverse and representative sample of Chinese adolescents. Participants were recruited from 48 middle schools distributed across 29 provinces, autonomous regions and municipalities in China, covering a wide geographical and demographic spectrum (Fig. 1). This extensive sampling strategy resulted in a substantial initial sample size of 22,428 participants. Inclusion criteria for participants were: (1) adolescents aged 12–18 years, (2) currently enrolled in middle school, and (3) capable of independently completing the survey without assistance. Exclusion criteria included: (1) previous diagnosis of psychiatric disorders, (2) current use of psychoactive medication, or (3) a history of substance abuse. The data collection period was from August 20 to October 10, 2024. Due to errors and omissions in responses, 610 questionnaires were discarded, resulting in 21,818 valid questionnaires, yielding a response rate of 97.28%. Full sociodemographic characteristics for included participants have been sorted into the Table 1.

**Table 1.** **Demographic and basic characteristics of participants**.

Demographic variables		Number (%)	Demographic variables		Number (%)
Age	15.04 ± 1.801		Parents’ highest education level	Primary school or below	2141 (9.81%)
Gender	Male	9978 (45.73%)		Middle school	8664 (39.71%)
	Female	11,448 (52.47%)		High school or technical school	5993 (27.47%)
	Missing values	392 (1.8%)		University or college	4111 (18.84%)
Family patterns	Two-parent family	18,427 (84.46%)		Graduate or above	743 (3.41%)
	Single-parent family-father custody	1023 (4.69%)		Missing values	166 (0.76%)
	Single-parent family-mother custody	1031 (4.73%)	Subjective family economic status	Poor	2514 (11.52%)
	Blended family	813 (3.73%)		Average	18,301 (83.88%)
	Other	455 (2.09%)		Wealthy	727 (3.33%)
	Missing values	69 (0.32%)		Missing values	276 (1.27%)
Frequency of returning home	Daily	9653 (44.24%)	Objective family economic status	Below 60,000 CNY	8195 (37.56%)
	Weekly	8512 (39.01%)		60,000–150,000 CNY	8266 (37.89%)
	Monthly	2941 (13.48%)		150,000–300,000 CNY	3650 (16.73%)
	Each semester	596 (2.73%)		Above 300,000 CNY	1136 (5.21%)
	Missing values	116 (0.53%)		Missing values	571 (2.62%)

Note: CNY, Chinese Yuan. 60,000 CNY ≈ 8322 USD, 150,000 CNY ≈ 20,805 USD, 300,000 CNY ≈ 41,610 USD.

### 2.2 Measurements 

#### 2.2.1 Perceived Parental Involvement Scale

The perceived parental involvement (PPI) Scale [27], is designed to assess adolescent perceptions of parental involvement. The questionnaire consists of 21 items in four dimensions of involvement: emotional, social, life and academic. Specifically, emotional involvement includes nine items, social involvement five items, life involvement four items and academic involvement three items. It uses a Likert five-point scale, where one indicates “almost never” and five indicates “always”. Higher total scores indicate greater perceived parental involvement. Psychometrically, the PPI shows excellent internal consistency, with an overall Cronbach’s α coefficient of 0.922, indicating high reliability. The inter-dimension correlation coefficients range from 0.399 to 0.580, while the correlations between each dimension and the total score range from 0.664 to 0.890, suggesting good construct validity. The structural validity indices meet psychometric standards (comparative fit index (CFI): 0.955, tucker-lewis index (TLI): 0.948, χ^2^/df: 6.917, root mean aquare error of approximation (RMSEA): 0.054 and standardized root mean square residual (SRMR): 0.081). Additionally, in the study sample Cronbach’s α is 0.929, further confirming questionnaire reliability.

#### 2.2.2 Center for Epidemiologic Studies Depression Scale

The Chinese revised version of the center for epidemiologic studies depression scale (CES-D) is mainly used for screening depressive symptoms. It consists of 20 items rated on a zero to three scale: Zero: “rarely or none”, one: “sometimes”, two: “half the time or often”, and three: “most of the time or continuously”. Items four, eight, twelve and sixteen are reverse-scored. The scoring criteria are: Total score ≤16: No depression; sixteen < total score ≤ nineteen: Possible depressive mood; total score ≥20: Indicates differing levels of depressive mood. Higher scores suggest a greater likelihood of the subject experiencing depressive mood. Cronbach’s α for this scale is 0.903, indicating good internal consistency [28].

### 2.3 Data Analysis 

For missing data, a two-step approach was adopted. It is based on the proportion of data missing for each variable. Specifically, if a variable had less than 20% missing data, mean substitution was applied. Variables with more than 20% missing data were excluded from the analysis. Raw data were entered and initially analyzed using SPSS (IBM, Inc., version 29.0.1.0, Armonk, NY, USA), R (R Foundation for Statistical Computing, version 4.3.1, Vienna, Austria) and Mplus (Muthén & Muthén, version 8.0, Los Angeles, CA, USA). To evaluate the impact of varying dimensions of parental involvement on depression, analysis of inferential statistics was performed with linear mixed-effect models (LMM) built with the lme4 package in R. A random effect for clustering of adolescents within regions was accounted to capture variability between groups. Missing data were removed in Sociodemographic characteristics. The LMMs for the outcome included one model with PPI total Score as a fixed effect and a separate model for each of the four involvement dimensions: emotional, social, life and academic. All models were adjusted for age, gender, family patterns, frequency of returning home and family socioeconomic status. SES encompasses the highest education level of parents, subjective family economic status and family economic status. By building upon the LMM model, regression coefficients (β values) and their 95% confidence intervals were calculated to assess the differential effects of parental involvement levels on depressive mood among adolescents. To further evaluate the models, ANOVA was employed to compare different model fits, allowing for the determination of the most predictive model for depressive symptoms. This comprehensive approach ensured robust evaluation of the relationship between parental involvement and adolescent depression.

Mplus was used for latent profile analysis (LPA), with missing values imputed by mean substitution. Data analysis proceeded as follows: (1) Independent sample *t*-tests and χ^2^-square analyses were conducted to examine demographic differences in parental involvement perception and depression. (2) LPA was utilized to explore the joint patterns among adolescent perceptions of parental involvement (emotional, social, life academic involvement) and family socioeconomic status (highest parental education, objective family economic level, subjective family economic level). Before LPA, variables were standardized using Z-score transformation. The optimal model for different combination patterns was estimated based on three criteria: (1) Lower values of the Akaike Information Criterion, bayesian information criterion (BIC) and sample size adjusted BIC (aBIC) indicate better model fit; (2) Significant *p*-values in the Lo Mendell-Rubin Likelihood Ratio Test and Bootstrap Likelihood Ratio Test suggest that a *k*-class model fits better than a *k*-1 class model; (3) An entropy score closer to unity indicates a higher probability of accurate classification of individuals. Model selection also considered parsimony and interpretability. The present study should acknowledge the shortcoming that mean substitution assumes that missing data are missing completely at random, which may not always be the case. To address this limitation, sensitivity analyses were conducted by comparing results from mean substitution with those obtained using multiple imputation methods. We found the consistency of results across these approaches, suggesting that the impact of missing data on the final analysis was minimal. Despite this case, expanding these findings should bear in caution for potential biases introduced by missing data imputations. Finally, based on the LPA results, ANOVA was used to analyze differences in depression across different combinations of categories.

## 3. Results 

The prevalence of depressive symptoms among adolescents was distributed as follows: 12,255 individuals (56.17%) reported no depression, 1871 individuals (8.57%) exhibited suspected depressive mood and 7692 individuals (35.26%) demonstrated a certain level of depressive mood. Perceived parental involvement was assessed using a standardized scale, yielding a mean score of 64.92 (SD = 17.425, N = 21,818).

### 3.1 PPI Significantly Predicts Depression in Adolescents 

**Fig. 2.**
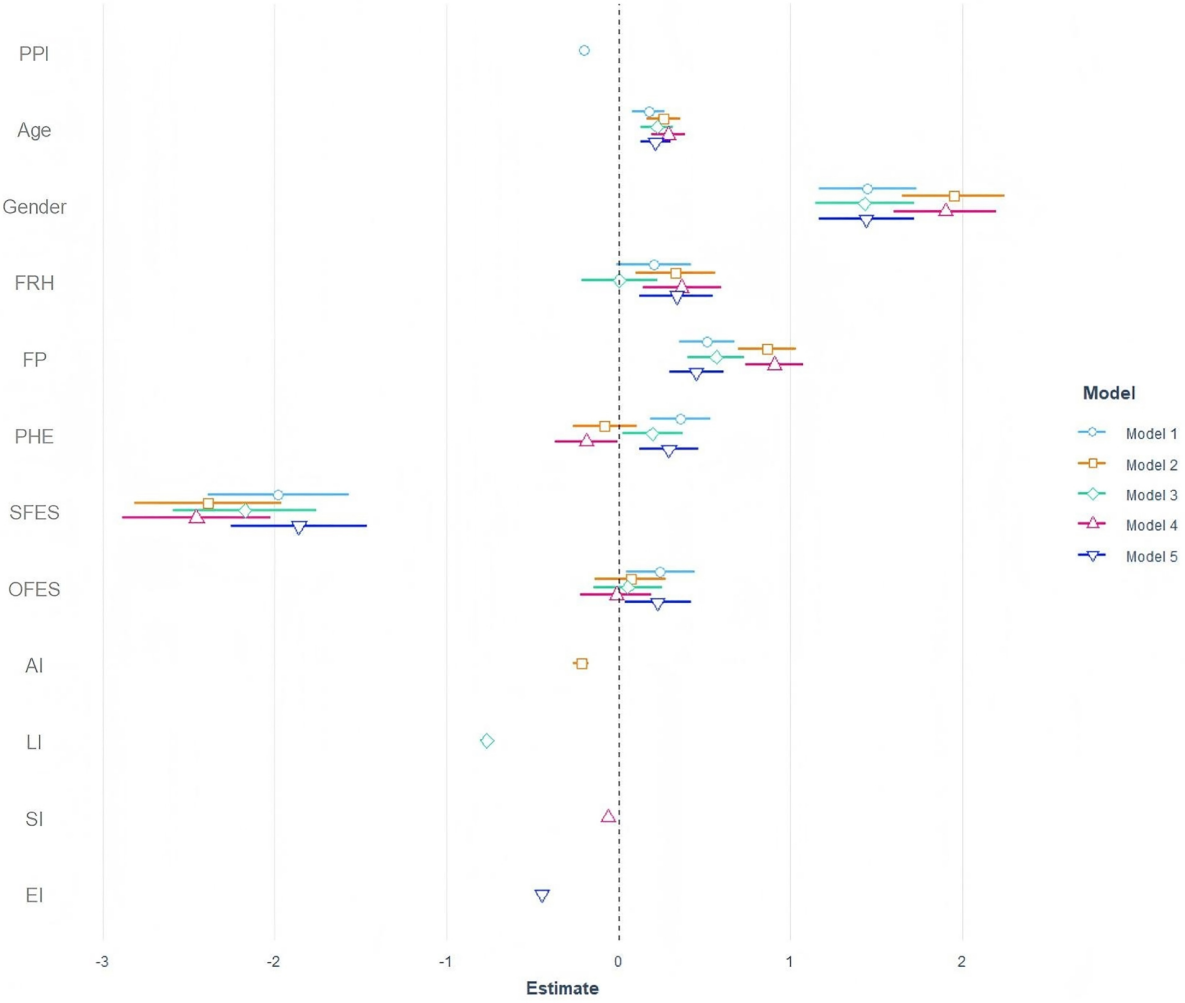
**Predictive effects of PPI and sociodemographic factors on adolescent depression: LMM estimates**. Note: FRH, frequency of returning home; FP, family patterns; PHE, parents’ highest education level; SFES, subjective family economic status; OFES, objective family economic status; AI, academic involvement; LI, life involvement; SI, social involvement; EI, emotional involvement.

This study used a LMM to analyze the predictive power of perceived parental involvement and its sub-dimensions on depression levels. Statistical adjustment was made for sociodemographic variables such as age, gender, family socioeconomic status (including parental highest education level, subjective family economic status and family economic status) and regional origin by using the linear mixed-effect model (LMM). Here, regional origin was defined as the participant provincial administrative living area in the geospatial distribution of mainland China. Results indicate that the total score of perceived parental involvement (β = –0.20, *p* < 0.001), as well as sub-dimensions including life involvement (β = –0.77, *p* < 0.001), academic involvement (β = –0.22, *p* < 0.001), social involvement (β = –0.06, *p* < 0.001), and emotional involvement (β = –0.45, *p* < 0.001), all negatively predicted CES-D scores. This suggests that increasing these forms of involvement may effectively reduce depression levels. Among all models, model five, which examines the impact of emotional involvement on adolescent depression, demonstrated the best fit indices, with a marginal R^2^ of 0.166 and a conditional R^2^ of 0.204 (see Table 2, Fig. 2).

**Table 2.** **LMM results for predicting CES-D scores based on PPI and its sub-dimensions**.

	CES-D			Random effects			N	Marginal R^2^	Conditional R^2^
	β	95% CI	*p*	σ^2^	τ_00_	ICC			
Model 1	–0.20	–0.21 to –0.19	<0.001	105.35	5.18_Region_	0.05	20,312	0.118	0.159
Model 2	–0.22	–0.26 to –0.17	<0.001	114.77	6.21_Region_	0.05	20,312	0.033	0.082
Model 3	–0.77	–0.81 to –0.73	<0.001	107.60	4.80_Region_	0.04	20,312	0.096	0.134
Model 4	–0.06	–0.09 to –0.03	<0.001	115.18	6.05_Region_	0.05	20,312	0.029	0.077
Model 5	–0.45	–0.46 to –0.43	<0.001	99.31	4.79_Region_	0.05	20,312	0.166	0.204

Note: Model 1, examines the effect of perceived parental involvement on adolescent depression; Model 2, examines the effect of academic involvement on adolescent depression; Model 3, examines the effect of life involvement on adolescent depression; Model 4, examines the effect of social involvement on adolescent depression; Model 5, examines the effect of emotional involvement on adolescent depression. β, beta coefficient; CI, confidence interval; *p*, *p*-value; σ^2^, residual variance; τ_00_, random intercept variance; ICC, intraclass correlation coefficient; Marginal R^2^, proportion of variance explained by the fixed effects alone; Conditional R^2^, proportion of variance explained by both fixed and random effects, providing an overall measure of model fit; N, sample size; CES-D, center for epidemiologic studies depression scale; PPI, perceived parental involvement; LMM, linear mixed-effect models.

ANOVA results showed that the model five it was significantly superior to others (akaike information criterio (AIC): 151,138, BIC: 151,225, Log-Likelihood (logLik): –75,558, deviance: = 151,116, χ^2^: 3013.7, *p* < 0.0001, see Table 3). Therefore, compared to the total involvement score or other dimensions, the degree of adolescent’s perceived parental involvement in the emotional domain is significantly greater than the other predictors of depression levels.

**Table 3.** **Comparison of model fit indices for predicting adolescent depression**.

	AIC	BIC	logLik	Deviance	χ²	*p*
Model 1	152,338	152,426	–76,158	152,316		
Model 2	154,081	154,168	–77,029	154,059	0.0	0.000
Model 3	152,766	152,853	–76,372	152,744	1315.0	0.000
Model 4	154,152	154,239	–77,065	154,130	0.0	0.000
Model 5	151,138	151,225	–75,558	151,116	3013.7	0.000

Note: AIC, akaike information criterion; BIC, bayesian information criterion; logLik, log-likelihood; χ^2^, chi-square statistic.

### 3.2 Identification of Three Latent Profiles of Family Socioeconomic and PPI

**Fig. 3.**
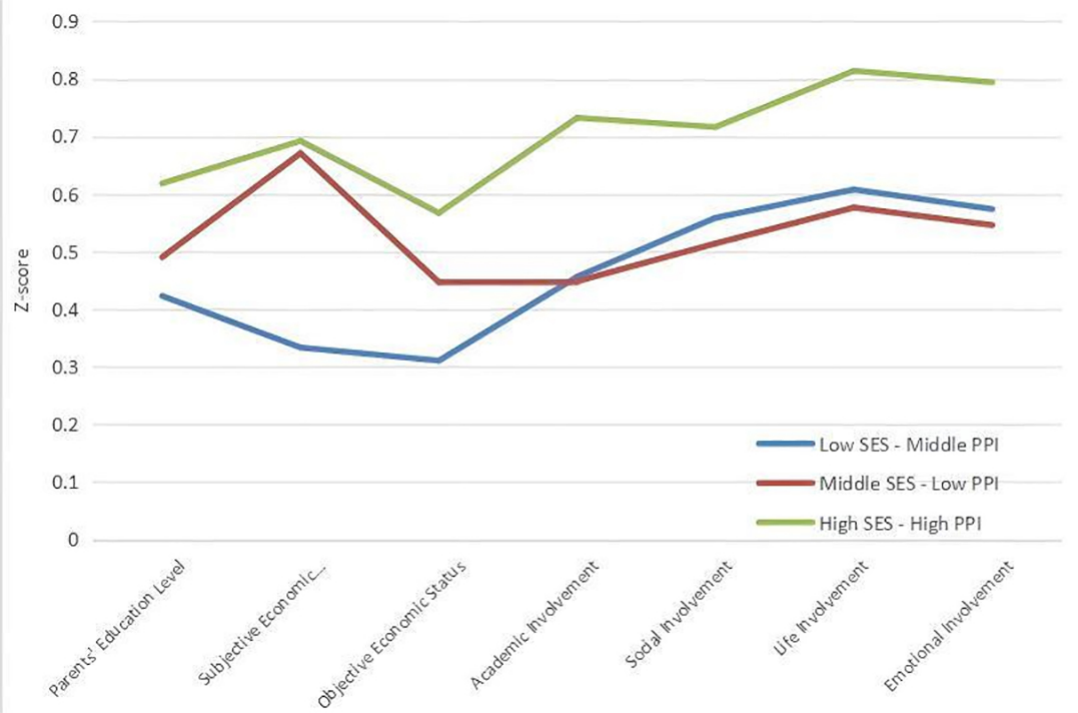
**Profile plot of family factors**. Note: SES, socioeconomic state.

LPA analysis initially used family factors as observed variables, starting from a single-class model and increasing sequentially. The fit indices for each model are given in Table 4. Results indicate that as the number of classes increases, AIC, BIC and aBIC values gradually decrease, while entropy values remain above 0.8, suggesting classification accuracy greater than 90%. For these reasons, the three-class model is accepted as it balances model simplicity and interpretability and is the best-fitting model (Fig. 3). In this model, Class 1 is characterized by the lowest socioeconomic status and average parental involvement, termed “Low SES-Middle PPI” (11.82%). Class 2 exhibits balanced socioeconomic status and lowest parental involvement, termed “Middle SES-Low PPI” (53.18%). Class 3 has the highest socioeconomic status and parental involvement, termed “High SES-High PPI” (35.00%).

**Table 4.** **Fit indices for latent profile analysis of family factors**.

	AIC	BIC	aBIC	Entropy	LMR (*p*)	BLRT (*p*)	Proportion of latent classes
1	640,119.348	640,230.620	640,186.129				
2	620,297.668	620,472.525	620,402.609	1.000	0.000	0.000	11.81%/88.19%
3	**605,224.348**	**605,462.789**	**605,367.450**	**0.830**	**0.000**	**0.000**	**11.82%/53.18%/35.00%**

Note: Bold indicates the best fitting model and fit indices. aBIC, sample-size adjusted BIC; LMR, lo-mendell-rubin likelihood ratio test; BLRT, bootstrap likelihood ratio test; Entropy, information entropy.

**Fig. 4.**
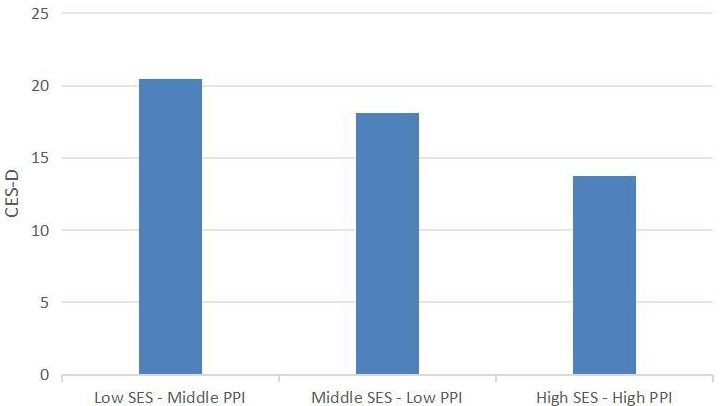
**Differences in depression scores across family categories**. Note: CES-D, center for epidemiologic studies depression scale.

Differences in depression scores among adolescents from different family factor models are illustrated in Fig. 4. The results of the ANOVA analysis indicate significant differences in depression scores across the categories (F = 757.917, *p* < 0.001). Post-hoc analyses revealed that the Low SES-Middle PPI group exhibited the highest depression scores, followed by the Middle SES-Low PPI group, while the High SES-High PPI group demonstrated the lowest depression scores (*p* < 0.05). The ANOVA analysis demonstrated statistically significant differences in adolescent depression scores across family factor models, with higher scores in the Low SES-Middle PPI group compared to the High SES-High PPI group.

## 4. Discussion 

This study found that perceived parental involvement and various of its dimensions significantly predict adolescent depression levels, with emotional involvement showing the strongest negative predictive effect. This highlights the crucial role of emotional support in alleviating depressive symptoms. Additionally, LPA revealed that different family types significantly influence adolescent depressive symptoms, particularly the family SES.

Results indicate a relatively high prevalence of depressive symptoms among adolescents, with 35.26% exhibiting some degree of depression. This finding is consistent with global trends, which highlight that emotions play a crucial role in the onset and progression of adolescent depression. A study has shown that adolescents who struggle with emotional regulation, including difficulty in identifying and managing negative emotions, are at a higher risk of developing depressive symptoms [29]. Furthermore, emotional dysregulation, which involves the inability to control or adapt emotional responses to stress, is strongly linked to increased vulnerability to depression during adolescence [30]. The relationship between negative emotions and depression can also be amplified by social factors, such as peer relationships and family dynamics, making emotional experiences a critical factor in understanding adolescent depression [31, 32]. These findings underscore the importance of addressing emotional health in interventions aimed at preventing and treating depression in adolescents.

Findings also support that both the overall perceived parental involvement and its distinct dimensions uniquely predict adolescent depression levels. Emotional involvement, in particular, emerged as the most influential dimension, showing the largest negative prediction of adolescent depression. Emotional involvement from parents plays a crucial role in adolescent mental health by enhancing psychological resilience [33], fostering self-esteem [34, 35] and aiding identity formation [36]. Secure emotional bonds, as explained by attachment theory, provide adolescents with a foundation for healthy emotional development, enabling them to develop effective coping strategies and exhibit lower stress levels [37]. This support buffers against environmental stressors such as peer pressure and academic challenges, reducing depressive symptoms even in stressful situations [5]. Cultural variations also influence the impact of emotional involvement, with collectivist cultures placing greater emphasis on family cohesion [38]. Research indicates that parental support in academics can enhance adolescent self-efficacy and sense of achievement, thereby reducing depression risk [5]. However, when compared to emotional involvement, the impact of academic involvement may depend more on adolescent personal values regarding academic success and cultural context. In some cultures, excessive academic pressure might have adverse effects, increasing anxiety and depression risks [39]. Parental involvement in daily life, such as participating in family activities or daily decision-making, provides adolescents with stability and a sense of belonging. This form of involvement is closely linked to adolescent overall well-being and mental health [40].

However, the impact of life involvement may vary depending on family structure and parental time investment, particularly in dual-income families where such involvement might be limited [41]. Parental engagement in adolescent social life, such as encouraging peer interactions or community involvement, helps adolescents build healthy social networks, reducing feelings of loneliness and depressive symptoms [42]. However, excessive social control or intervention may lead to adolescent resistance and social stress.

The superior fit indices of the emotional involvement model underscore its predictive power, suggesting that interventions aimed at enhancing emotional connections between parents and adolescents could be particularly effective in reducing depression. By prioritizing emotional support, parents create a nurturing environment that addresses the emotional needs of adolescents, thereby alleviating depressive symptoms more effectively than focusing solely on academic or social aspects. Overall, the study emphasizes the multifaceted nature of parental involvement and its critical role in adolescent mental health. While all dimensions contribute to reducing depression, emotional involvement stands out as having the greatest impact, highlighting the need for targeted strategies that strengthen emotional bonds within families. These findings align with existing literature while also highlighting the diversity and complexity of parental involvement across different cultural and socioeconomic contexts.

The present study identified three distinct family profiles—“Low SES-Middle PPI”, “Middle SES-Low PPI” and “High SES-High PPI”. This provides valuable insights into how socioeconomic status and parental involvement interact to influence adolescent mental health. Adolescents in the “Low SES-Middle Parental Involvement” group face the highest risk of depression, despite moderate levels of parental involvement. Key finding worthy to note is to demonstrate the complex interplay between economic stress and parental involvement. While moderate parental involvement may provide some emotional support, it appears insufficient to counteract the significant negative impacts of low SES, such as resource scarcity and chronic stress. Adolescents in this group may experience heightened feelings of helplessness and insecurity due to unmet material needs, which could amplify depressive symptoms. Furthermore, the mismatch between parental efforts an unmet adolescent expectation for support may exacerbate frustration and emotional distress. Future research should explore what effect targeted interventions may have in such circumstances [43, 44, 45]. In the “Middle SES-Low Parental Involvement” group, adolescents exhibit moderate depression scores, indicating that even with relatively favorable economic conditions, a lack of parental involvement can still negatively impact mental health. A middle socioeconomic status typically implies that families have certain economic resources, but if parents are not emotionally engaged, adolescents may feel isolated and lack a sense of belonging. This emotional detachment can result in a lack of support and guidance when facing challenges, thereby increasing the risk of depression [46, 47]. This underscores the critical role of parental involvement in promoting adolescent mental health, particularly in terms of emotional support and guidance. The “High SES-High Parental Involvement” group reports the lowest depression scores, reflecting the dual advantage of abundant material and emotional support. High socioeconomic status is often associated with better educational and extracurricular opportunities, which can help adolescents develop skills and interests, enhancing their confidence and sense of achievement. Additionally, high levels of parental involvement provide emotional support and may foster better academic and social development, creating a safe and supportive environment for adolescents [48]. Such an environment helps reduce the risk of depression, as adolescents can access ample support and resources when facing life challenges [49]. These findings emphasize the need to consider both economic and emotional factors within families when assessing and intervening in adolescent depression. Developing personalized support strategies for adolescents from different family backgrounds may be more effective, particularly in resource-limited contexts where enhancing parental involvement could serve as a crucial intervention point.

In summary, this study reveals the multifaceted nature of parental involvement and its critical role in adolescent mental health. While all dimensions of parental involvement contribute to reducing depression, emotional involvement stands out as the most impactful. This highlights the need for targeted strategies that strengthen emotional bonds within families. The identification of distinct family profiles—“Low SES-Middle PPI”, “Middle SES-Low PPI” and “High SES-High PPI”—provides valuable insight into how socioeconomic status and parental involvement interact to influence adolescent mental health. Findings highlight the diversity and complexity of parental involvement and adolescents depression across Chinese cultural contexts.

Despite the robustness of the findings, this study has several limitations. First, the cross-sectional design limits the ability to infer causality between parental involvement and adolescent depression. Longitudinal studies are needed to establish causal relationships and examine changes over time. Second, the reliance on self-reported measures may introduce bias, as adolescent perceptions of parental involvement might not fully capture the full reality of parental behavior. Future research should incorporate multi-informant approaches, including parental reports and observational data, to establish a more comprehensive understanding. Additionally, cultural and contextual factors were not extensively explored here. Future research should investigate how cultural norms and values influence parental involvement and its impact on adolescent mental health across diverse populations. Finally, while the study identified key family profiles, further exploration of other potential moderating variables, such as peer influence and school environment, could enrich understanding of the complex dynamics affecting adolescent depression.

## 5. Conclusions 

In conclusion, this study highlights the significant role of perceived parental involvement, particularly emotional involvement, in predicting adolescent depression levels. The findings emphasize the importance of fostering strong emotional connections within families to enhance adolescent mental health. By addressing both economic and emotional factors, tailored interventions can be developed to effectively support adolescents from diverse family backgrounds. As the prevalence of depressive symptoms among adolescents remains high, such insights are crucial for informing policy and practice aimed at promoting mental well-being. Future research should continue to explore the intricate interplay of familial, cultural and contextual factors to develop comprehensive strategies for preventing and alleviating adolescent depression.

## Availability of Data and Materials

The raw data supporting the conclusions of this article will be made available by the authors, without undue reservation.

## Declaration of AI and AI-Assisted Technologies in the Writing Process

This manuscript has undergone editing and enhancement using ChatGPT 4o (OpenAI) to improve the clarity, logic, flow, and phrasing of the writing, particularly in the Introduction and Discussion sections. While the AI tool has contributed to refining the text for better readability and coherence, it is important to note that all scientific content and contexts included in this work were originally drafted by human authors. The authors retain full responsibility for the research findings and interpretations presented in the manuscript. The use of AI was solely to assist in elevating the quality of expression without altering the original scientific ideas or data.
